# Use of Telehealth in Substance Use Disorder Services During and After COVID-19: Online Survey Study

**DOI:** 10.2196/25835

**Published:** 2021-02-08

**Authors:** Todd Molfenter, Nancy Roget, Michael Chaple, Stephanie Behlman, Olivia Cody, Bryan Hartzler, Edward Johnson, Maureen Nichols, Patricia Stilen, Sara Becker

**Affiliations:** 1 Center for Health Enhancement System Studies University of Wisconsin-Madison Madison, WI United States; 2 Center for the Application of Substance Abuse Technologies University of Nevada-Reno Reno, NV United States; 3 New York State Psychiatric Institute Division on Substance Use Disorders Columbia University Irving Medical Center New York, NY United States; 4 Alcohol & Drug Abuse Institute University of Washington Seattle, WA United States; 5 National Center for Primary Care Morehouse School of Medicine Atlanta, GA United States; 6 Addiction Research Institute Steve Hicks School of Social Work The University of Texas at Austin Austin, TX United States; 7 School of Nursing and Health Studies University of Missouri Kansas City, MO United States; 8 Center for Alcohol and Addiction Studies Brown University Providence, RI United States

**Keywords:** COVID-19, substance use disorders, technology acceptance model, telehealth

## Abstract

**Background:**

Social distancing guidelines for COVID-19 have caused a rapid transition to telephone and video technologies for delivering treatment for substance use disorders (SUDs).

**Objective:**

This study examined the adoption of these technologies across the SUD service continuum, acceptance of these technologies among service providers, and intent of providers to use these technologies after the pandemic. Additional analysis using the validated technology acceptance model (TAM) was performed to test the potential applications of these technologies after the pandemic. The study objectives were as follows: (1) to assess the use of telehealth (telephone and video technologies) for different SUD services during COVID-19 in May-June 2020, (2) to assess the intended applications of telehealth for SUD services beyond COVID-19, (3) to evaluate the perceived ease of use and value of telehealth for delivering SUD services, and (4) to assess organizational readiness for the sustained use of telehealth services.

**Methods:**

An online survey on the use of telephonic and video services was distributed between May and August 2020 to measure the current use of these services, perceived organizational readiness to use these services, and the intent to use these services after COVID-19. In total, 8 of 10 regional Addiction Technology Transfer Centers representing 43 states distributed the survey. Individual organizations were the unit of analysis.

**Results:**

In total, 457 organizations responded to the survey. Overall, the technology was widely used; >70% (n>335) of organizations reported using telephone or video platforms for most services. The odds of the intent of organizations to use these technologies to deliver services post COVID-19 were significantly greater for all but two services (ie, telephonic residential counseling and buprenorphine therapy; mean odds ratio 3.79, range 1.87-6.98). Clinical users preferred video technologies to telephone technologies for virtually all services. Readiness to use telephone and video technologies was high across numerous factors, though telephonic services were considered more accessible. Consistent with the TAM, perceived usefulness and ease of use influenced the intent to use both telephone and video technologies.

**Conclusions:**

The overall perceived ease of use and usefulness of telephonic and video services suggest promising post–COVID-19 applications of these services. Survey participants consistently preferred video services to telephonic services; however, the availability of telephonic services to those lacking easy access to video technology is an important characteristic of these services. Future studies should review the acceptance of telehealth services and their comparative impact on SUD care outcomes.

## Introduction

COVID-19 has led to rapid virtualization of health care services, as in-person care needs to be delivered using telephone or video technologies. For example, nearly half of Medicare-covered primary care consultations were delivered virtually in April 2020 compared to 0.1% delivered before the pandemic in February 2020 [1]. Although Seema Verma (administrator, Centers for Medicare & Medicaid Services) stated that telehealth “will never replace the gold-standard of in-person care” [2], experts have acknowledged that rapid uptake of telehealth since the onset of COVID-19 may transform the health care system.

Undoubtedly, COVID-19 has accelerated the use of technology to deliver substance use disorder (SUD) services. However, technology usage for health care services has been gradually trending upward before COVID-19 [3,4] owing to five factors. First, smartphone ownership in the United States increased from 35% in 2011 to 81% in 2019 [5], thereby increasing access to online health resources. Second, mobile phone ownership has increased among low-income individuals. A recent study reported that 94% of homeless adults had access to a mobile phone, suggesting new opportunities to increase access to care in underserved populations [6]. Third, provider interest and the adoption of patient-centered technologies have strengthened [7]. Fourth, the feasibility and effectiveness of technology to deliver SUD treatment services have increased with the advent of both asynchronous internet-based technologies [3,8] and synchronous telephone- [9-11] and video-based [12-14] therapies. Fifth, increasing evidence is available regarding telehealth across health care services, and telehealth has increased patient access, adherence, and retention to care services [15]. Several preliminary studies have reported that telehealth yields equivalent outcomes to in-person care [16-18].

Despite these emerging trends, the actual adoption of telephonic and video SUD services has been slow [7,13]. Emerging data suggest that organization type (eg, health system, specialty treatment clinics, etc) [19] and location (eg, suburban, rural, urban, etc) [20] can potentially influence the readiness to use these technologies. Other organization-level factors influencing the readiness to adopt these technologies include financial resources for their deployment and perceptions of their ease, customization, clinical efficacy, and ability to enhance workflow [21-23]. Time for staff training and acceptance, technology accessibility, and access to information technology experts are additional key considerations [20,22,24]. In addition, patients’ perceptions and preferences toward technology are also important. Patients’ acceptance of these technologies affects the patient-clinician relationship and can influence staff acceptance of patient care technologies [25,26]. The perceived ease of use and value of these technologies influence both staff and patient acceptance of them, which, in turn, influence decisions regarding their continued use [27-29].

This study aimed to fill an existing knowledge gap by surveying the use of telephone and video technologies in SUD services, while simultaneously assessing the projected use of these technologies beyond COVID-19 and evaluating the readiness of organizations to use them. Furthermore, this study investigated whether the technology acceptance model (TAM)—which has linked the intent to use a technology and actual technology use in numerous trials [30]—can predict the intent to use these technologies. We hypothesized that (1) >50% of organizations use telephone and video technologies, (2) the odds of increased post–COVID-19 use of these technologies among organizations would be significant, and (3) consistent with the TAM, the perceived usefulness of telephone and video technologies would mediate the effect of their ease of use on the intent to use them. Owing to the scarcity of data in these areas, we did not propose *a priori* hypotheses on preferences for telephone vs video technologies.

## Methods

### Study Design

We developed an online survey (Multimedia Appendix 1) to measure the use of telephone and video technologies for delivering a specific set of SUD services [7], gauge the intent to use telephonic and video services after COVID-19, and explore the perceived readiness to use telephonic and video services, using TAMs previously developed by Gustafson et al [21] and Davis et al [31]. Designated regional Addiction Technology Transfer Centers (ATTCs) distributed the survey in their respective regions. Substance Abuse and Mental Health Services Administration (SAMHSA)-funded ATTCs support the workforce for addiction treatment and recovery. These regional ATTCs correspond to the 10 regional offices of the US Department of Health and Human Services. In total, 7 of 10 regional ATTCs representing 43 states partnered in this survey. The 3 nonparticipant regional ATTCs represented the remaining 7 states; these ATTCs declined participation, citing survey overburden due to other unrelated surveys in the field.

### Data Collection

The survey was distributed and data were collected from May 15 to August 31, 2020. Principal investigators at the regional ATTCs distributed survey links to SUD treatment and recovery support administrators or personnel (physicians, counselors, and peer recovery coaches). They used various methods to disseminate the survey and obtain a convenience sample as large and representative as possible, including the use of regional ATTC listservs and partnering with state policymakers to share the invitation widely. The survey questionnaire contained 79 questions. The respondents could change their answers before submitting the survey. Cookies were not used to identify unique users, but no incentive was provided to the respondents for completing the questionnaire, thus limiting the likelihood of duplicate survey submissions. Multiple responses from the same organization were averaged and accounted for in regression analysis. Only submitted surveys were analyzed. The University of Wisconsin’s institutional review board approved the survey distribution and the recruitment of study participants (approval number 2020-0551). All data were collected using REDCap, a secure web application [32].

### Survey Instruments

The survey included the following components and scales: organization type (ie, health system, opioid treatment programs, recovery community organizations, and specialty addiction treatment providers [nonopioid treatment programs]), organization location (ie, rural, small city, suburban, and urban), and organizational role of the respondents (ie, administrators and personnel providing treatment and recovery services, including counselors, physicians, and recovery coaches). Since rural individuals with SUDs are typically underserved [33] and are more markedly impacted by the opioid epidemic [34], and rural providers are more prone to adopt telehealth [20], the rural representativeness of the sample will be assessed using data from the National Treatment Center study [33].

For telehealth use, the survey assessed (1) the use of telephone and video technologies for the following services: screening and assessment, buprenorphine therapy, case management, intensive outpatient treatment, peer recovery support, regular outpatient treatment, and residential counseling sessions with binary yes/no variables; and (2) the projected intent to use telephonic and video services after implementing COVID-19 safety measures for these services, as per the following categories: “less than before,” “about the same,” “little more than before,” or “much more than before.”

The Organizational Readiness for Technology Use predictive tool developed by Gustafson et al [21] was used to assess dimensions of organizational readiness for the use of telephone and video technologies. Each item was evaluated using a 5-point Likert scale with endpoints of 1=“strongly disagree” and 5=“strongly agree.” The inventory assessed the perceived feasibility of reimbursement for the technology during and after COVID-19; access to information technology experts, clinical champions, and billing experts to support the use of these technologies; ease of technology integration into the workflow; staff, facilities, and equipment to promote the technology; leadership, staff, and patient support; technology accessibility and affordability; and staff training.

The technology acceptance survey included two subscales from the TAM [35,36]: ease of use and perceived usefulness. The ease of use scale assesses the ease of learning, customizing, and using the technology. Perceived usefulness assesses the extent to which the technology is perceived to enhance effectiveness, improve performance, increase productivity, and be useful. Items in these subscales were scored on 5-point Likert scales with endpoints ranging from “strongly disagree” to “strongly agree.” These subscales were used in conjunction with the projected intent to use determined by the survey participants.

### Data Analysis

Frequency distributions were used to describe organizational characteristics (setting and type), participant job descriptions, the use of telephone and video technologies for different SUD services, and the intent to use these technologies to deliver various services post COVID-19. In 3 regions, an overall survey response rate was calculated using a query on unique organizations identified from regional ATTC databases. For Regional ATTCs that lacked the capacity to conduct this query (n=5), SAMHSA’s Treatment Episode Database was used to estimate the available number of total SUD treatment organizations in that region’s states. The rural representativeness of the sample was calculated using data from the National Treatment Center Study [33], in accordance with a robust literature base indicating that rural organizations have been disproportionately affected by the opioid epidemic [34] and are more likely to adopt telehealth [20]. We used linear mixed-effects models (LMMs) to investigate differences in the intent to use telephone and video technologies based on organization location or setting and the job functions of the survey respondents, accounting for multiple respondents within the same organization. These models are expressed using the following equation:


Intent_ij_ = β_0 _+ β_1_X + u_i_ + ε_ij _**(1)**

where X denotes either the organization location, setting, or job function of the survey respondent; u_i_ is the random intercept for organizations, and ε_ij_ is the within-organization random error. 

Composite scores for the intent to use telephone and video technologies were generated by averaging those of the intent to use services after the implementation of COVID-19 safety measures across the different SUD services considered herein. To compare the odds of using telephone vs video technologies for the different post–COVID-19 services, a generalized LMM was used. These analyses compared the odds of reporting “more use”/“little more use” of these technologies post COVID-19 to those of reporting “about the same”/“little less” for telephone and video technologies post COVID-19. These models are expressed using the following equation: 


Response_ij_ = β_0 _+ β_1_Technology + u_i_ + ε_ij _**(2)**

where u_i_ denotes the random intercept for organizations, and ε_ij_ is the within-organization random error. 

Variables determining organizational readiness for technology adoption were analyzed by comparing the scores for organizational readiness for the use of telephone and video technologies by using LMMs to investigate differences in factors between these technologies. These models are expressed using the following equation: 


Readiness_ij_ = β_0 _+ β_1_Technology + u_i_ + ε_ij _**(3)**

where u_i_ denotes the random intercept for organizations; and ε_ij_ is the within-organization random error. 

Lastly, the TAM data were analyzed through mediation analysis, which compared the perceived ease of use and perceived usefulness variables to the composite intent of use variables for telephonic and video services. These mediation analyses were conducted using linear regression models to predict the use of technology [36]. These models are expressed using the following equations: 


Future Intent = β_10 _+ β_11_Ease of Use + ε_1 _**(4)**

Perceived Ease of Use = β_20 _+ β_22_Perceived Usefulness + ε_2_ **(5)**

Future Intent = β_30 _+ β_33_Ease of Use + β_33_Perceived Usefulness + ε_3 _**(6)**

where ε is the error in the estimation of the intent and ease of use. All analyses were conducted using the lme4 package (RStudio).

## Results

The survey respondents represented 457 unique organizations from 43 states. The survey was distributed to an estimated 2785 organizations that provide SUD services, with an estimated return rate of 16%. A total of 92 (20.1%) organizations identified themselves as rural, which closely approximated that reported in the National Treatment Center study (19.9%). In total, 268 (58.6%) organizations provided specialty treatment (excluding opioid treatment programs), whereas 101 (22.2%) were health organizations.

**Figure 1:**
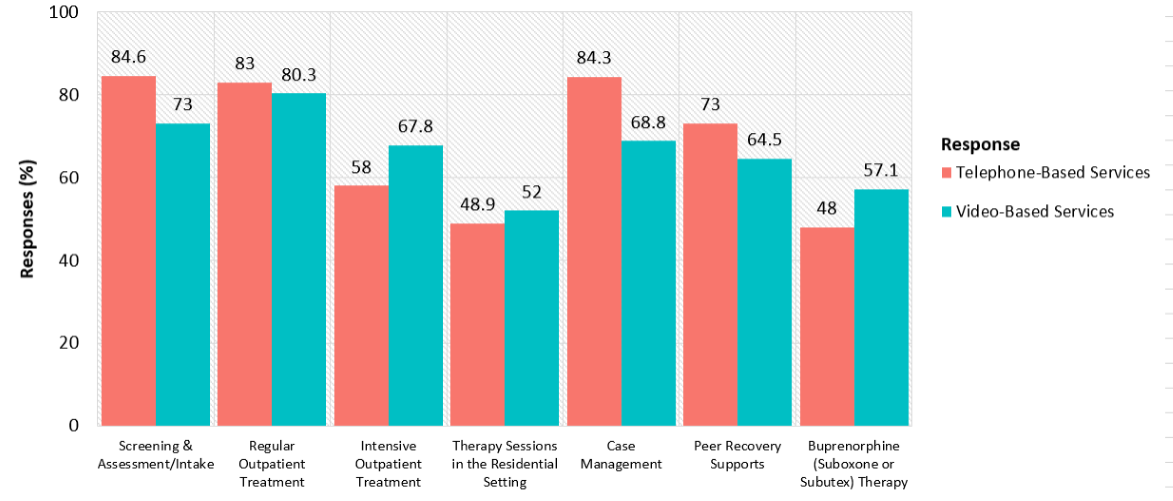
Rates of use of different telehealth services.

Figure 1 shows the current rates of using telephonic and video SUD services by service type. In total, 387 (84.6%) organizations used telephonic SUD services most frequently for screening and assessments. Furthermore, 367 (80.3%) organizations used video SUD services most frequently for regular outpatient treatment. We observed the most marked difference in the use of telephone (n=386 [84.4%] respondents) and video (n=314 [68.8%] respondents) technologies for case management.

As a first step in investigating future use, we examined whether the intent to use telephonic or video services beyond COVID-19 varied as a function of organization location or type or staff type. No significant differences resulted from the organization location (ie, rural, urban, suburban, or urban). Regarding organization type, the intent to use telephonic services post COVID-19 was significantly greater for health systems (mean 2.99; 95% Cl 2.66-3.32) than for specialty treatment sites (mean 2.77; 95% Cl 2.65-2.88) (*P*=.04) (Table 1). In total, among the survey respondents, 187 (38.7%) were administrators and 270 (61.3%) were personnel who provide treatment and recovery services, and no significant difference in the intent to use telephonic or video services post COVID-19 were observed between them (*P*=.51).

**Table 1:** Characteristics of the participating organizations (N=457).

Predictor			Organizations, n (%)		Future intent of using telephonic services					Future intent of using video services	
					Estimate (95% CI)		*P* value		Estimate (95% CI)		*P* value
**Organization setting**											
	Rural	92 (20.1)		2.83 (2.64 to 3.02)		<.001		2.97 (2.78-3.16)			<.001
	Small city	94 (20.5)		–0.02 (–0.28 to 0.25)		.90		–0.02 (–0.29 to 0.24)			.87
	Suburban	80 (17.4)		0.23 (–0.05 to 0.05)		.11		0.08 (–0.19 to 0.36)			.56
	Urban	191 (42)		–0.06 (–0.29 to 0.18)		.63		0.07 (–0.17 to 0.31)			.55
**Organization type**											
	Specialty treatment	268 (58.6)		2.77 (2.65 to 2.88)		<.001		3.04 (2.93 to 3.16)			<.001
	Health system	101 (22.2)		0.22 (0.01 to 0.44)		.04		–0.01 (–0.23 to –0.20)			.90
	Opioid treatment programs	47 (10.2)		0.14 (–0.15 to 0.42)		.35		–0.17 (–0.47 to –0.14)			.29
	Recovery community	41 (9)		0.22 (–0.11 to 0.55)		.19		–0.03 (–0.36 to –0.29)			.84
**Respondent job function**											
	Administrator	187 (41)		2.87 (2.72 to 3.01)		<.001		3.13 (2.99 to 3.28)			<.001
	Personnel providing treatment and recovery services	270 (59)		–0.06 (–0.25 to 0.12)		.51		–0.17 (–0.35 to –0.02)			.08

As shown in Table 2, all the SUD services had a positive odds ratio (OR) for the intent to use telephone or video technologies, reporting responses of “much more” or a “little more” after COVID-19 compared to those of “about the same” or a “little less” before COVID-19. The only exceptions were for nonsignificant ORs for using telephonic services in the residential setting or for buprenorphine therapy post COVID-19. In general, the odds of using video technology were greater than those of using telephone technology for all services, except for case management (OR 1.37, 95% CI 0.94-2.01; *P*=.10) and peer recovery services (OR 1.06, 95% Cl 0.70-1.61; *P*=.78, prospectively).

**Table 2:** Odds of using telehealth post COVID-19.

Service	Telephone technology			Video technology			Video vs telephone technologies	
	OR^a^ (95% CI)	*P* value	OR (95% CI)		*P* value	OR (95% CI)		*P* value
Screening and assessment/intake	3.06 (1.91-4.90)	<.001	5.49 (3.28-9.19)		<.001	1.79 (1.25-2.57)		.001
Regular outpatient treatment	4.01 (2.41-6.68)	<.001	6.98 (3.99-12.19)		<.001	1.74 (1.20-2.52)		.003
Intensive outpatient treatment	1.59 (1.02-2.48)	.04	3.85 (2.33-6.34)		<.001	2.42 (1.53-3.81)		<.001
Residential therapy sessions	1.87 (0.98-3.56)	.06	4.09 (2.04-8.22)		<.001	2.19 (1.26-3.83)		.006
Case management	3.74 (2.24-6.25)	<.001	5.12 (2.98-8.80)		<.001	1.37 (0.94-2.01)		.10
Peer recovery supports	4.06 (2.33-7.06)	<.001	4.31 (2.47-7.52)		<.001	1.06 (0.70-1.61)		.78
Buprenorphine (Suboxone or Subutex) therapy	1.18 (0.62-2.26)	.61	3.69 (1.81-7.55)		<.001	3.12 (1.68-5.81)		<.001

^a^OR: odds ratio.

Table 3 summarizes the organizational readiness for technology use measures. For telephonic services, three factors had the highest average rating on the 5-point Likert scale: (1) telephonic counseling is affordable to patients (3.83, 95% Cl 3.72-3.94), (2) our leadership supports the implementation of telephonic counseling (3.78, 95% Cl 3.67-3.89), and (3) most of our patients have access to telephonic counseling (3.78, 95% Cl 3.67-3.89); video services: (1) our leadership supports the implementation of video counseling (3.87, 95% Cl 3.75-3.98), (2) staff want video counseling to be sustained (3.72, 95% Cl 3.61-3.84), and (3) video counseling easily integrates into our workflow (3.66, 95% Cl 3.55-3.77) and is affordable to patients (3.66, 95% Cl 3.55-3.77).

Analysis of ORs for video vs telephonic services revealed several significant differences between these technologies (Table 3). Video services were considered less advantageous for the following factors: most of our patients can access the technology (–0.71, 95% Cl –0.83 to –0.59; *P*<.001), patients find that telephonic/video counseling is easy (–0.51, 95% Cl –0.63 to –0.40; *P*<.001), patients want telephonic/video counseling to be sustained (–0.17, 95% Cl –0.28 to –0.07; *P*=.001), and counseling is affordable to patients (–0.17, 95% Cl –0.28 to –0.05; *P*=.004). Video services were considered more advantageous for the following factors: there is a clinical champion for the promotion of counseling (0.29, 95% Cl 0.17-0.40; *P*<.001), and we anticipate being adequately reimbursed for the services we provide with counseling after COVID-19 (0.11, 95% Cl 0.01-0.21; *P*=.04).

**Table 3:** Organizational readiness for using telephone and video technologies.

Factor	Telephone technology, OR^a^ (95% CI)	Video technology, OR (95% CI)	Video vs telephone technologies	
			OR (95% CI)	*P* value
Most of our patients can access telephonic/video technologies	3.78 (3.67 to 3.89)	3.07 (2.95 to 3.18)	–0.71 (–0.83 to –0.59)	<.001
Our leadership supports the implementation of telephonic/video counseling	3.78 (3.67 to 3.89)	3.87 (3.75 to 3.98)	0.09 (–0.01 to 0.19)	.09
Patients find telephonic/video counseling is easy to use	3.75 (3.64 to 3.85)	3.23 (3.12 to 3.34)	–0.51 (–0.63 to –0.40)	<.001
Patients want telephonic/video counseling to be sustained	3.68 (3.56 to 3.79)	3.50 (3.39 to 3.62)	–0.17 (–0.28 to –0.07)	.001
Staff has been properly trained in telephonic/video counseling	3.35 (3.23 to 3.48)	3.39 (3.26 to 3.51)	0.03 (–0.08 to 0.14)	.59
Staff, facilities, equipment, job descriptions, and policies are in place for sustaining telephonic/video counseling	3.43 (3.32 to 3.55)	3.53 (3.41 to 3.65)	0.09 (–0.01 to 0.20)	.09
Staff want telephonic/video counseling to be sustained	3.66 (3.55 to 3.77)	3.72 (3.61 to 3.84)	0.06 (–0.05 to 0.18)	.30
Telephonic/video counseling easily integrates into our workflow	3.63 (3.52 to 3.74)	3.66 (3.55 to 3.77)	0.03 (–0.08 to 0.14)	.62
Telephonic/video counseling is affordable to patients	3.83 (3.72 to 3.94)	3.66 (3.55 to 3.77)	–0.17 (–0.28 to –0.05)	.004
There is a clinical champion for the promotion of telephonic/video counseling	3.14 (3.01 to 3.26)	3.42 (3.30 to 3.54)	0.29 (0.17 to 0.40)	<.001
We anticipate being adequately reimbursed for the services we provide with telephonic/video counseling after COVID-19	3.27 (3.14 to 3.39)	3.38 (3.25 to 3.50)	0.11 (0.01 to 0.21)	.04
We are adequately reimbursed for the services we provide with telephonic/video counseling during COVID-19	3.32 (3.20 to 3.44)	3.38 (3.26 to 3.50)	0.06 (–0.04 to 0.16)	.23
We have the billing expertise to support use of telephonic/video counseling in our organization	3.55 (3.43 to 3.66)	3.59 (3.47 to 3.71)	0.04 (–0.05 to 0.14)	.38
We have the information technology expertise to support the use of telephonic/video counseling in our organization	3.53 (3.41 to 3.65)	3.53 (3.42 to 3.65)	0.01 (–0.10 to 0.12)	.90

^a^OR: odds ratio.

**Figure 2:**
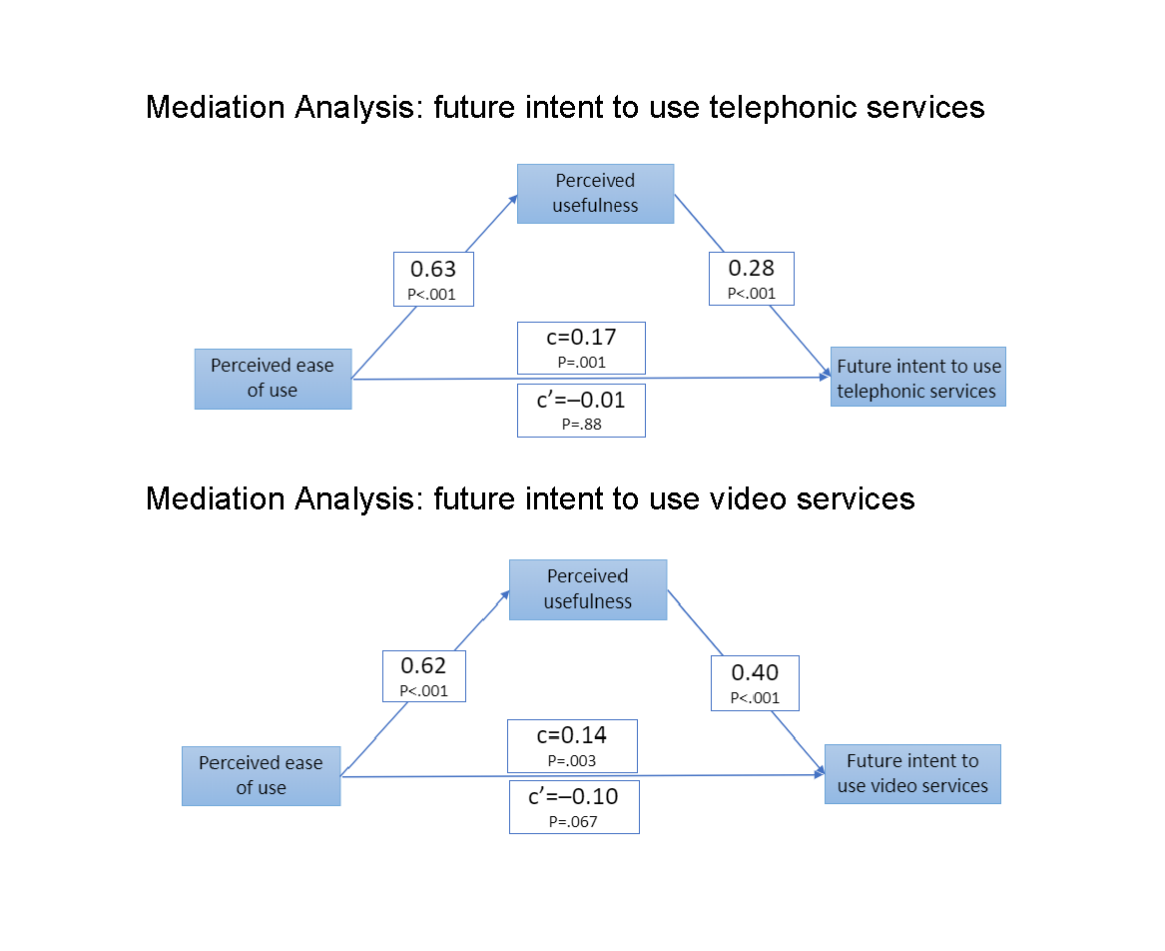
Technology acceptance model for telephonic services and video substance use disorder services.

Figure 2 presents the findings of the TAM. Specifically, these analyses tested the perceived usefulness of these technologies as a mediator of the effects of the ease of their use on the intent to use them. Separate analyses were conducted for telephonic and video services. On mediation analysis for telephonic services, perceived ease of use was significantly associated with future intent of use (*P*=.001). Inclusion of perceived usefulness to the model resulted in significant associations between perceived ease of use and perceived usefulness (*P*<.001) and between perceived usefulness and future intent of use (*P*<.001). The association between perceived ease of use and future intent of use was not significant (*P*=.88), indicating complete mediation. Mediation analysis revealed similar associations for the intent to use video services, wherein the path between perceived ease of use and future intent to use was significant (*P*=.003). This path was no longer significant (*P*=.07) upon including perceived usefulness in the model (Figure 2).

## Discussion

### Principal Findings

This study surveyed administrators and personnel from SUD treatment and recovery organizations to evaluate their current and projected use of telehealth for different SUD services during the early months of the COVID-19 pandemic. We examined key concepts for telephonic and video services separately, including the current use of technology, intent to use these technologies post COVID-19, and organizational readiness for using these technologies. We hypothesized that organizations would report high usage rates (>50%) of the current telephone and video technologies across a range of SUD services and would report significant odds of intending to increase their usage of these technologies post COVID-19. Furthermore, we conjectured that consistent with the TAM, perceived usefulness of technology would mediate the effects of ease of use on the intent to use these technologies. We did not propose *a priori* hypotheses about which SUD services would be most conducive to telehealth or whether differences would emerge between telephonic and video services.

Regarding the use of telehealth services during the survey, consistent with our hypotheses, most organizations used various services. Screening was the most common telephonically delivered service, whereas outpatient treatment was the most common video-delivered service.

These results are encouraging, since numerous studies have suggested that drug and alcohol screens can be administered telephonically with high levels of reliability and validity, and that outpatient treatment delivered via videoconferencing has comparable effectiveness to in-person care [37].

Regarding the future intent to use these services, consistent with our hypotheses, organizations reported significant odds of increasing their use of telehealth and video services after COVID-19. The two services for which respondents did not anticipate using telephonic services were buprenorphine therapy and residential counseling. For both of these services, respondents reported their willingness to use video-based services, suggesting their receptiveness to using technology in general, but they expressed specific concerns about using the telephone. The reticence to prescribe buprenorphine telephonically could reflect various factors, including provider mistrust and stigma toward patients with opioid use disorders, as well as concerns about diversion [38]. Use of video services was viewed more favorably, compared to telephonic services, for most SUD services.

Regarding organizational readiness for technology use, systematic differences emerged between telephone and video technologies. Relative to video services, respondents perceived telephonic services as more advantageous in terms of access, ease of use, affordability, and ease of sustainability. By contrast, video services were perceived as more valuable in terms of the likelihood of reimbursement and having the support of a clinical champion. Respondents preferred video services to telephonic services for all but two services. These findings are consistent with those of previous studies reporting that video-based counseling is associated with higher patient satisfaction but is substantially more expensive and not necessarily associated with superior levels of abstinence [39]. With the emergence of new videoconferencing tools, telephonic counseling would likely still have value owing to its simplicity, affordability, and reach, particularly among patients with limited access to video-based technologies.

Finally, on performing TAM analysis, the mediation model supported our hypothesis that the perceived usefulness of technology would mediate the association between the ease of use and the intent to use. This model emphasizes the critical role of the perceived usefulness in the adoption and current use of technologies. The survey outcomes were encouraging, in that SUD program administrators and personnel perceived the use of telephone and video technologies as useful during and after COVID-19.

### Limitations

However, our results should be considered within the context of several limitations. First, participants were recruited through convenience sampling of administrators and leaders contacted by principal investigators of regional ATTCs via email. Hence, the sampling methods might have resulted in a selection bias, such that individuals most comfortable with technology were most likely to complete the electronic survey. Furthermore, this sampling approach limits direct comparisons between participants who completed the survey and those who did not, though we could estimate a response rate and examine the rural representativeness of the sample on the basis of publicly available nationwide data. Second, even though the survey respondents represented 43 states and a range of organizational settings, the present findings based on a limited response rate may not be extrapolated to the general population. Third, patient-level data were not collected during sampling, thus limiting the representativeness of this sample and nationwide SUD organizations on the basis of the characteristics of the patients they serve.

### Conclusions

Nonetheless, our results provide a promising outlook toward the use of telephonic and video services after COVID-19. Regarding the future applications of telehealth, this study suggests that the rapid transition to widespread use of telephonic and video services—necessitated almost overnight owing to COVID-19 stay-at-home orders and social distancing guidelines—is associated with high levels of provider receptivity to telephone technology. Clinicians perceived video services more appealing but telephonic services more accessible, suggesting that both channels play a role in the delivery of SUD services. Respondents’ perceptions of the continued use of telehealth post COVID-19 and their general readiness to use it support the positive outlook toward the role of telehealth in SUD services. Future studies are required to review the acceptance of these different service delivery approaches and their impact on care outcomes.

## Appendix

Multimedia Appendix 1 Telehealth survey questions.

## Abbreviations

ATTC: Addiction Technology Transfer Center
LMM: linear mixed-effects model
OR: odds ratio
SAMHSA: Substance Abuse and Mental Health Services Administration
SUD: substance use disorder
TAM: technology acceptance model
