# Supplementary figures and images for: Use of Telehealth in Substance Use Disorder Services During and After COVID-19: Online Survey Study
Source: JMIR Ment Health. 2021 Feb 8;8(2):e25835. doi: 10.2196/25835 (PMC7895293; doi:10.2196/25835)

**Appendix**: Use of Telehealth via Telephone/Video Survey Questions


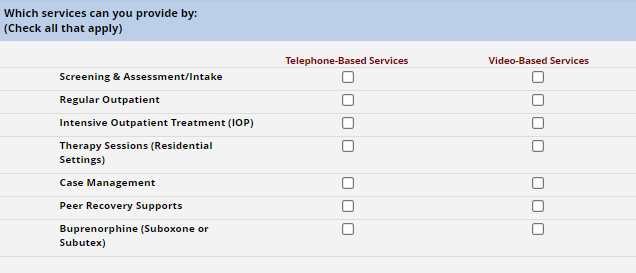


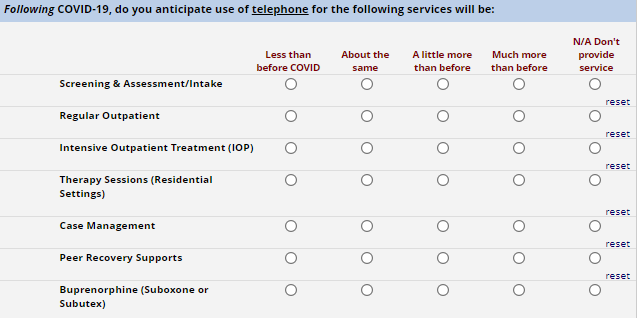


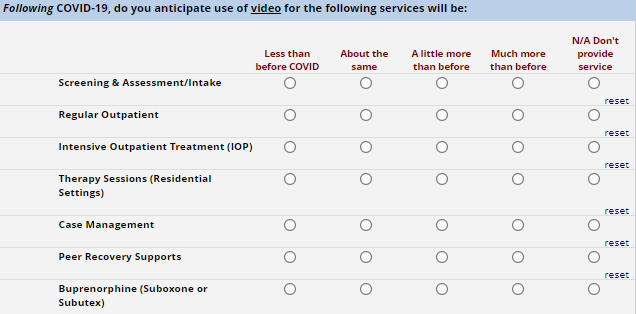

Supplement: Multimedia Appendix 1 [file mental_v8i2e25835_app1.docx]
